# External fixation of fractures of the distal radius: A randomized comparison of the Hoffman compact II non-bridging fixator and the Dynawrist fixator in 75 patients followed for 1 year

**DOI:** 10.1080/17453670902807433

**Published:** 2009-02-01

**Authors:** Yngvar Krukhaug, Stein Ugland, Stein A Lie, Leiv M Hove

**Affiliations:** ^1^Department of Orthopedic Surgery, Haukeland University HospitalHelse-Bergen HF, BergenNorway; ^2^Department of Orthopedic Surgery, Soerlandet Hospital HFKristiansandNorway; ^3^Department of Health, University Research BergenBergenNorway; ^4^Department of Surgical Sciences, University of BergenBergenNorway

## Abstract

**Background and purpose** External fixators allowing movement during fracture healing are commonly used for treatment of unstable distal radius fractures. The dynamic Dynawrist fixator with the distal pins in metacarpal bone may avoid fixation problems in comminuted fractures and may reduce the risk of nerve injury. We compared anatomical and functional outcome for the well-established Hoffmann compact II non-bridging fixator and for the Dynawrist fixator.

**Patients and methods** 75 patients with unstable distal radius fractures were randomized to treatment with either the Hoffman compact II fixator (the H-group) or the Dynawrist fixator (the D-group). Anatomical and functional variables were recorded preoperatively, postoperatively, and at 6, 12, 24, and 52 weeks. Pain was assessed using the VAS score and function was assessed using DASH score.

**Results** Postoperatively, radial tilt, inclination, and radial length all improved statistically significantly in both groups. At time of removal of the fixators, the H group had superior volar radial tilt. At the 52-week follow-up, there were no statistically significant differences between the groups regarding anatomical variables. At 6 weeks, flexion was greater in the D group but at 12, 24, and 52 weeks flexion was similar in the two groups, as were the other wrist and forearm movements. There were no statistically significant differences between the groups according to VAS and DASH scores. 3 nerve injuries occurred in the H group and 1 in the D group (p = 0.4), all of which were transient.

**Interpretation** The Dynawrist bridging but dynamic fixator gives radiographic and functional outcome similar to that of the Hoffman II compact non-bridging fixator.

## Introduction

Different types of external fixation are used for fractures of the distal radius. These include static bridging fixators (Solgaard 1989, Ludvigsen et al. 1997), non-bridging fixators (Jenkins et al. 1987, McQueen 1998), and bridging dynamic fixators (Clyburn 1987, Sommerkamp et al. 1994).

Non-bridging fixators, with pins only in the radius, permit wrist movement, but the distal pins may be difficult to insert in comminuted fractures and they may also injure the superficial branch of the distal radial nerve. To avoid these problems, we developed a bridging but dynamic device, the Dynawrist fixator (Hove et al. 1999).

We compared the radiographic and functional outcome of the well-established non-bridging Hoffman II compact fixator (McQueen et al. 1999) with that of the Dynawrist fixator.

## Patients and methods

This was a consecutive, randomized series of patients with unstable fractures of the distal radius, suitable for non-bridging external fixation. All patients had an AO-type A3 fracture, with an intact volar cortex of the distal fragment of at least 1 cm. The patients were treated at one of 2 hospitals between January 2004 and December 2005.

Patients who were included were at least 18 years old, and had one or more of the following fracture deformities: more than 10 degrees of dorsal angulation and/or radial shortening of more than 2 mm compared to the uninjured wrist at the initial radiography. Patients treated with closed reduction and cast were included if they had radiocarpal malalignment of more than 5 mm, a dorsal angulation of more than 5 degrees, or a shortening of the radius of more than 2 mm at the 10-day follow-up. 32 patients were initially treated with plaster cast, which failed to maintain the reduction and required surgery. If surgery was delayed for more the 14 days after the fracture, the patient was not included. Patients who were suffering from dementia or psychiatric diseases, or who had a history of previous fracture in one of the wrists were also excluded.

75 patients (64 women) were included. Their mean age, which was similar in both groups, was 62 (20–92) years. 39 patients had fractures of the non-dominant wrist. Mean time from injury to surgery was 4 (0–14) days. All patients were treated with closed reduction; after randomization, 37 patients were treated with the Hoffman II fixator (the H group) and 38 patients were treated with the Dynawrist fixator (the D group).

The Regional Ethics Committee approved the study and informed consent was obtained from all patients.

### Operative technique

**Figure 1.**
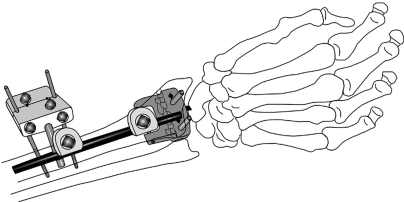
The Hoffman II compact non-bridging external fixator.

In the H group, 2 longitudinal incisions (1 cm long) were made on the dorsum of the wrist, one on either side of Lister’s tubercle. 2 longitudinal incisions were made in the extensor retinaculum, taking precautions to avoid injury to the extensor pollicis longus tendon. 2 Apex pins were then placed parallel to the joint surface in the distal radial fragment, from dorsal to volar, engaging the volar cortex. 2 pins were placed in the radial shaft with open technique. The fracture was reduced using the distal pins as levers (Figure 1).

**Figure 2.**
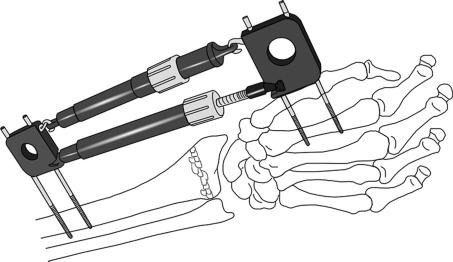
The Dynawrist external fixator.

In the D group, 2 Apex pins were placed in the second metacarpal bone parallel to the palm. By open placement technique and with the use of a guide, 2 pins were placed in the radial shaft, parallel to the palm with the forearm in neutral rotation. The springs were adjusted until the radial length was acceptable and the pins in the shaft were parallel to those in the second metacarpal bone (Figure 2).

Fluoroscopy was used with both techniques peroperatively, to confirm the placement of the Apex pins and the reduction of the fractures. The surgery was performed in plexus anesthesia. Postoperative radiographs were taken within 24 h of surgery. The operated arm was kept elevated for the first 48 h. All patients had compression bandages around the Apex pins, covering the incisions. The mean fixation time, which was similar in both groups, was 43 (33–59) days.

### Evaluation

The patients were followed up at 6, 12, 26, and 52 weeks after the operation. An independent observer performed all postoperative measurements.

### Anatomical assessment

**Figure 3.**
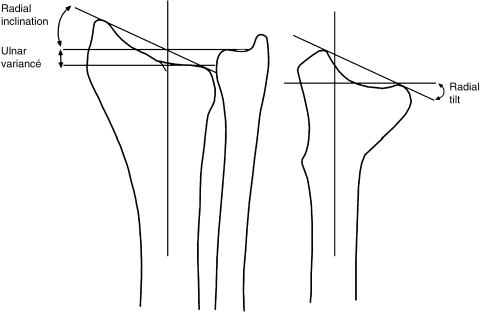
Standard description of the measurements of radiological deformities.

Pre- and postoperatively, at the time of removal of the fixator, and 1 year after the injury, standard anteroposterior and lateral radiographs were taken of the injured wrists. They were compared to the radiographs of the uninjured side taken preoperatively. Radial tilt, ulnar variance, and radial inclination before and after fixation were measured according to standard descriptions (van den Linden and Ericson 1981) (Figure 3).

### Functional assessment

Range of motion (ROM; flexion, extension, radial deviation, ulnar deviation, supination, and pronation) of the injured wrist and forearm was measured at 6 weeks (the time of removal of the fixator), and at 12, 26, and 52 weeks. The difference in movement was calculated by measuring ROM of the uninjured side and then subtracting the ROM of the injured side.

The total upper extremity function was assessed by self-evaluation, using a Scandinavian translation of the DASH score (Hudak et al. 1996, Atroshi et al. 2000). We recorded the degree of pain using VAS score.

2 patients died after the 6-week follow-up, and 2 patients did not want to attend after the 6-week follow-up. These 4 patients were in the H group.

### Statistics

A sample size calculation showed that in order to show a difference with a 5% significance level and 80% power, 35 individuals in each group would be needed for radial tilt as outcome, while more than 8,000 individuals would be needed with inclination as the outcome.

The patients were randomly allocated into one of the two groups using closed envelopes, which were opened after the patients had given their informed consent.

The 95% confidence intervals were calculated as mean values ± 1.96 times the standard errors. To account for the repeated measures, we used a mixed model for repeated measures with an autoregressive correlation structure, to estimate the mean differences between the two products for all time points. The mean differences, 95% confidence intervals, and p-values for comparisons of the mean values at 6-, 12-, 26-, and 52-week follow-ups when evaluating functional measures, and postoperatively, at time of removal, and at 1-year follow-up when evaluating anatomical measures, were calculated using dummy variables for each of the time points in the mixed models. Parameters for the baseline measures were not included in the models dealing with the anatomical variables. However, the baseline measures were accounted for in the calculation of the standard errors and hence the 95% confidence intervals and the p-values.

The statistical package SPSS for Windows, release 14, was used for the analyses. We considered p-values less than 0.05 to be statistically significant.

## Results

Complete data were collected from 71 patients, and partly from 4 patients who dropped out (2 due to death and 2 because of severe health-related problems).

### Anatomical assessment

Preoperatively, the median radial tilt was 29 degrees of dorsal angulation in the H group and 32 in the D group. Postoperatively, the median tilt was 8 degrees of volar angulation in the H group and 2 degrees volar in the D group (p = 0.002).

At the time of removal of the fixators, there was still a statistically significant difference in radial tilt: 9 degrees of volar angulation in the H group and 4 degrees in the D group (p = 0.04). At 1 year, the difference was no longer statistically significant. For the other anatomical variables, no statistically significant differences were found (Table 1).

**Table 1.** Anatomical assessments, mean (95% CI)

	Group	Preoperatively n = 75	Postoperatively n = 75	At removal n = 75	At 1 year n = 71
Radial tilt (degrees)	Hoffman Dynawrist Mean difference	29 (25–34) dorsal 32 (27–36) dorsal	8 (6–10) volar 2 (0–5) volar 5 (1–9)	9 (7–11) volar 4 (2–6) volar 4 (1–8)	8 (6–10) volar 4 (2–6) volar 4 (0–8)
Ulnar variance (mm)	Hoffman Dynawrist Mean difference	4 (3–4) 4 (3–5)	0 (0–1) 0 (-1–0) 1 (0–2)	1 (0–2) 0 (-1–0) 1 (0–2)	1 (0–2) 0 (0–1) 1 (0–2)
Radial inclination (degrees)	Hoffman Dynawrist Mean difference	17 (15–19) 18 (16–20)	23 (22–24) 23 (22–24) 0 (-1–2)	23 (22–24) 24 (23–24) 0 (-2–2)	23 (21–24) 23 (21–24) 0 (-2–3)

### Functional assessment

At 6 weeks, the mean loss of flexion was 34 degrees in the H group and 24 degrees in the D group (p = 0.001). At the other times, the differences between the groups were not statistically significant. There were no statistically significant differences between the groups concerning loss of extention, radial and ulnar deviation, supination, or pronation at the different times (Table 2).

**Table 2.** Functional assessments: mean (95% CI) loss of movement in the injured wrist compared to the uninjured wrist. Values given represent degrees

	Group	6 weeks n = 75	12 weeks n = 75	26 weeks n = 73	52 weeks n = 71
Flexion	Hoffman Dynawrist Mean difference	35 (28–40) 24 (20–28) 10 (5–16)	18 (9–28) 15 (9–20) 4 (-3–10)	8 (4–12) 9 (7–12) -2 (-8–3)	3 (-1–8) 8 (5–10) -5 (-11–6)
Extention	Hoffman Dynawrist Mean difference	43 (35–49) 42 (37–47) 1 (-5–6)	23 (14–31) 25 (19–29) -1 (-8–7)	11 (7–15) 10 (7–13) 1 (-4–7)	9 (6–12) 4 (1–6) 5 (-1–11)
Radial deviation	Hoffman Dynawrist Mean difference	12 (7–18) 12 (8–15) 0 (-5 – 5)	6 (-3–14) 6 (1–10) 2 (-4 – 8)	0 (-4–5) -1 (-4–2) 2 (-3–7)	2 (-2–5) -1 (-3–1) 2 (-3–7)
Ulnar deviation	Hoffman Dynawrist Mean difference	13 (9–18) 12 (8–16) 2 (-3–6)	7 (-2–16) 10 (4–16) 0 (-7–6)	5 (1–9) 2 (-1–5) 3 (-2–8)	2 (-1–5) 2 (-1–5) 0 (-5–5)
Supination	Hoffman Dynawrist Mean difference	31 (22–42) 30 (23–37) 3 (-5 – 10)	13 (3–22) 9 (4–14) 4 (-5–13)	7 (3–10) 10 (6–12) -1 (-9–6)	4 (2–7) 8 (5–11) -4 (-11–4)
Pronation	Hoffman Dynawrist Mean difference	15 (9–23) 20 (14–27) -5 (-10–1)	4 (-2–10) 6 (1–12) -2 (-10–5)	1 (-1–4) 2 (0–5) -1 (-2–1)	0 (-2–2) -1 (-7–5) 1 (5–7)

There were no statistically significant differences in mean values of the VAS score between the groups at any time (data not shown).

At 52 weeks, the mean (CI 95%) DASH score was 9 (3–14) in the H group and 13 (8–20) in the D group.

### Complications

Injuries of the superficial branch of the radial nerve occurred in 3 patients in the H group and 1 in the D group (p = 0.4, Fisher’s exact test, 2-sided). The symptoms were transient and disappeared within 6 months. Superficial pin-track infections occurred in 9 patients in the H group and in 9 patients in the D group. There were no deep infections and no patients developed a complex regional pain syndrome.

## Discussion

Several external fixators that allow early exercise have been developed to prevent joint stiffness and shorten the period of rehabilitation (Hove et al. 1999, Slutsky 2007). Clyburne (1987) introduced what he called the first dynamic external fixator. It was assumed to reduce the disability associated with unstable fractures of the distal radius by allowing early joint movement. He used an external, wrist-bridging fixator with a ball-joint design. Today, a similar principle is used in an external fixator produced by Orthofix (Frykman et al. 1989). Sommerkamp et al. (1994) showed, however, that such ball-joint fixators are inferior to static fixators because the ball joint is not situated in the anatomical center of rotation; thus, joint movement will dislocate the fracture.

Some years later, McQueen (1998) introduced the “non-bridging” concept. She concluded in her randomized study that non-bridging external fixation was superior to bridging external fixation, regarding both anatomical and functional outcome. However, Atroshi et al. (2006) could not confirm these findings. Furthermore, the non-bridging technique has some limitations; a minimum of 1 cm of the volar cortex in the distal fragment must be intact, the operative technique is more demanding, and there is a risk of damaging the extensor tendons. There is also possibly increased risk of infection in the tendon sheets, the fracture zone, or the wrist joint as compared to the situation with bridging fixators.

In the bridging but dynamic Dynawrist fixator, the center of joint rotation is not disturbed (Hove et al. 1999). Like the non-bridging external fixators, it has the advantage of allowing early joint mobilization despite the fact that the distal pins are placed distal to the joint, the fracture zone, the extensor tendons, and tendon sheets. This reduces the risk of the abovementioned complications associated with non-bridging external fixation, as described above. Furthermore, Dynawrist can be used in more comminuted fractures, as it does not rely on a distal intact volar cortex of a certain size.

In recent years, the popularity of volar fixed-angle plates has increased—with promising early results (Rozental et al. 2003, Musgrave and Idler 2005, Chung et al. 2006). This open technique may introduce a new set of surgical complications. The current literature offers no evidence to support the use of internal rather than external fixation in the treatment of unstable fractures of the distal radius (Margaliot et al. 2005, Ochman et al. 2006). Until studies with a high level of evidence to the contrary are published, external fixators retain their place in the treatment of fractures of the distal radius.

We found the same anatomic and functional outcome in patients treated with the Dynawrist bridging but dynamic fixator and in patients treated with the non-bridging Hoffman II fixator. Since the surgical technique is simpler and the indications for application are wider, the Dynawrist fixator seems to be a good alternative to the Hoffman II fixator.
